# The Geometry of Suspicion: Visual Exploration Patterns in Email Phishing Detection

**DOI:** 10.3390/jemr19030060

**Published:** 2026-06-01

**Authors:** Francesco Di Nocera, Lorenzo Arciulo, Giorgia Tempestini, Pierpaolo Zivi, Giulio Errico, Fabio Ferlazzo

**Affiliations:** 1 Department of Planning, Design, and Technology of Architecture, Sapienza University of Rome, 00196 Rome, Italy; lorenzo.arciulo@uniroma1.it (L.A.); giorgia.tempestini@uniroma1.it (G.T.); 2 Department of Psychology, Sapienza University of Rome, 00185 Rome, Italy; pierpaolo.zivi@uniroma1.it (P.Z.); giulio.errico@uniroma1.it (G.E.); fabio.ferlazzo@uniroma1.it (F.F.)

**Keywords:** phishing, eye tracking, visual exploration, cybersecurity, attention, nearest neighbor index

## Abstract

This study examined visual exploration strategies in phishing-email detection by integrating conventional AOI-based eye-tracking measures with a complementary scene-based indicator, the Nearest Neighbor Index (NNI), to capture the global spatial organization of fixations. Thirty-two volunteers completed an email-classification task involving 106 static email stimuli; data from 30 participants were included in the final analyses. For each stimulus, participants judged whether the email was authentic or phishing, allowing for the computation of eye-tracking metrics across Signal Detection Theory classification outcomes. Concerning the NNI, the results showed that the spatial distribution of fixations was higher for suspicious than for non-suspicious emails, indicating a broader visual exploration pattern under higher task demands. More importantly, correct and incorrect responses differed reliably: hits were associated with more dispersed and regular fixation patterns, whereas false alarms were associated with more clustered scanning; misses showed a descriptively similar tendency that did not survive correction for multiple comparisons. Participants also responded faster when correct than when incorrect. When cybersecurity awareness (CAIN) was included as a mean-centered covariate, the primary effects of Signal and Outcome on NNI and decision time remained significant, indicating that the experimental effects are robust to individual differences in cybersecurity knowledge. However, CAIN did not emerge as a reliable predictor of eye-tracking measures within these models, suggesting that its role operates more at the level of classification performance than moment-by-moment gaze organization.

## 1. Introduction

Phishing is a form of cyberattack in which attackers create fraudulent websites or messages to obtain a victim’s sensitive information. Hackers commonly use fake emails and fake websites for this purpose. In email phishing, the user receives a message that appears to come from a trusted source, such as a bank or a government agency, and is prompted to click a link that is intended to steal personal or sensitive information.

Notwithstanding the widespread dissemination of information about phishing and the large number of studies devoted to this phenomenon, phishing attacks continue to be highly effective and remain one of the most successful forms of cybercrime. According to the Anti-Phishing Working Group [1], phishing remained a high-volume global threat in 2025, with approximately 3.8 million phishing attacks observed over the course of the year, highlighting its persistent and widespread impact.

The success of phishing suggests that many deceptive messages contain cues that are either overlooked, given too little weight, or evaluated too late during the inspection process; therefore, understanding how users visually scan malicious emails is essential for identifying which elements attract attention, which are neglected, and how these patterns of inspection may contribute to correct or incorrect judgments.

Eye tracking has become a key method for studying how users visually inspect phishing emails because gaze metrics provide process-level evidence about ongoing response allocation during evaluation tasks. Authors typically interpret these data under the assumption that fixations are a proxy for attention and processing, and that gaze measures align closely in time with evaluation processes [2,3]. In phishing-email research, eye tracking is used to complement outcome measures such as accuracy, perceived trustworthiness, and response intentions by showing how users visually inspect an email while they emit selection responses (e.g., “phishing” vs. “legitimate”). This focus is also supported by the view that email phishing should be studied separately from web phishing, since the tactics and detectable cues differ across media, and eye-tracking research on phishing emails is still more limited than work on phishing websites [2,4].

### 1.1. AOI-Based Approaches and Their Limitations

Methodologically, the most common approach is to define Areas of Interest (AOIs) that correspond to meaningful parts of the email, such as the header or sender, the body, URLs or links, and attachments, and then measure inspection through fixation-based and visit-based metrics. A representative example is provided by Pietrantonio et al. [5], who divide emails into header, body, and URL AOIs and compute measures such as total visit duration, total fixation duration, fixation count, visit count, coverage, and scanpath order to describe inspection strategies during phishing-identification tasks. Consistent with this broader trend, a recent review of gaze-based and EEG-based phishing research highlights the frequent use of fixation metrics and AOI time measures across experimental paradigms [3,5,6]. However, the literature also notes that experimental choices can introduce interpretive constraints: phishing materials sourced online may conflate multiple techniques (e.g., threat and urgency), and screenshots can limit interaction compared to real email clients, potentially shaping fixation and dwell patterns. Relatedly, AOI classification itself can be a limitation: AOIs may sometimes support “correct” decisions for the wrong reasons, and very small AOIs can be sensitive to eye-tracker error margins, requiring careful AOI design and (where needed) adjustment procedures.

Empirical findings from email-focused studies suggest that users often spend most of their viewing time on the email body, followed by the header, while the footer and signature receive relatively little attention during discrimination tasks. In the study by Pfeffel et al. [7], participants often began by inspecting the body or the header and moved from top to bottom, but after this initial pass, they tended to jump between regions and revisit them. This pattern suggests a shift from sequential scanning to more targeted, cue-focused checking. These observations are often considered important for phishing detection because the header can contain useful cues for identifying phishing, even though performance may still remain imperfect when users are explicitly told to distinguish phishing from legitimate emails [7].

Within this framework, individual differences, especially expertise and knowledge about cybersecurity, are repeatedly associated with differences in gaze strategy and, in some datasets, with better performance. Pietrantonio et al. [5] report that experts classified emails more accurately than non-experts and present preliminary evidence that experts spent more fixation time on the header, which they interpret as greater awareness of phishing indicators in that area. Similarly, Pfeffel et al. [7] find that their expert group paid relatively more attention to the header and attachments and needed less overall time to process the emails. They argue that both knowledge and processing time were important for successful recognition [5,7].

At the same time, several studies caution that greater attention to suspicious regions does not necessarily lead to correct detection. This is why gaze data should be analyzed together with behavioral outcomes rather than treated as a direct proxy for understanding. In line with this caution, the literature notes that dwell time on an AOI does not necessarily reflect the level of understanding of a security cue, and short glances do not necessarily indicate that an element was missed, meaning that the same gaze pattern can sometimes support different interpretations. For example, Ribeiro et al. [3] found that phishing emails produced longer fixation times and more fixations in the sender area than legitimate emails, but they did not observe significant correlations between eye-tracking measures across AOIs and identification accuracy. McAlaney and Hills [4] similarly found that participants looked at phishing-indicator regions more often than expected by chance and revisited them more frequently, yet they spent less total dwell time on those indicators than expected by chance. They also report that scanning time did not show a simple or direct relationship with trustworthiness judgments. Their findings further suggest that some indicator types, such as misspellings and threatening language, are linked to lower perceived trustworthiness, and that urgency and threat cues tended to be viewed before misspellings. This indicates that different cue types may differentially control responding even when the relation between gaze and outcomes is not straightforward [3,4].

Recent research has also expanded the range of cues under study by examining visually and psychologically manipulative design strategies in phishing emails, described as dark patterns that may bypass user scrutiny. In particular, Oh et al. [8] report that brand imitation and urgent language attracted the least visual attention, with significantly lower fixation duration, fixation count, and revisit count than other cues. This pattern supports the idea that persuasive but visually familiar elements may be systematically overlooked [8].

Studies that combine gaze traces with participants’ reported reasons for their judgements likewise suggest that protective behaviors may be applied only superficially, meaning that users may check cues without reliably extracting the information that is actually diagnostic. Zhuo et al. [9] report that looking at the sender was associated with lower phishing susceptibility. At the same time, they found that participants rarely hovered over links to inspect the actual URLs. When participants did check the URLs, they often did so through the browser, but there was no evidence that this strategy reliably reduced susceptibility in that specific context. More broadly, the literature highlights that both the contextual relevance of materials and the presence of salient design elements can influence how attention is allocated. This implies that gaze strategies observed in controlled tasks may change when messages align closely with users’ everyday goals and routines. They further argue that email-processing patterns and prior learning histories are important because early attention to salient cues and perceived relevance may shape both perceived trustworthiness and vulnerability during phishing encounters [9]. Taken together, these results emphasize a cue > attention > interpretation > action chain: users may perform “protective” checks yet remain vulnerable if they cannot interpret what they see as diagnostic of phishing.

Because spontaneous cue checking may not be sufficient, another line of research examines interface interventions designed to guide attention and better calibrate trust during email evaluation. Baltuttis and Teubner [2] study visual risk indicators in an eye-tracking experiment and report that these indicators affect both trust and response behavior, treating visual attention as central to the link between risk signaling, trust, and action. In this context, risk indicators refer to interface cues that explicitly signal a message’s suspected risk level (e.g., banners, labels, external-sender warnings, or risk scores), whereas trust indicators refer to cues that implicitly increase perceived legitimacy (e.g., brand logos, familiar layouts, or authoritative wording). At a more adaptive level, ADVERT (ADaptive Visual aids for Efficient Real-time security-assistive Technology) proposes an adaptive attention enhancement mechanism using attention signals/gaze-based features. This approach explicitly frames attention guidance as a phishing-prevention mechanism and connects attention measures to phishing-recognition performance [2,10,11].

Finally, the literature highlights several methodological threats, especially priming and limited ecological validity, that can affect both gaze patterns and performance in controlled phishing experiments. Pfeffel et al. [7] note as a limitation that participants were told in advance that the set included both phishing and legitimate emails, and they suggest more ecologically valid designs, such as embedding phishing messages within broader email-management tasks over time. Baltuttis and Teubner [2] similarly discuss sample bias and priming effects when participants are explicitly informed that the study concerns phishing detection, and they describe design choices intended to reduce these problems through broader recruitment and more general task framing. In line with this logic, Zhuo et al. [9] report that they did not disclose the true purpose of the study and did not tell participants that phishing emails would be present, in order to avoid biasing behavior in their scenario-based design [2,7,9].

### 1.2. Beyond AOIs: Global Organization of Visual Exploration

A specific contribution of the present study is to extend the analysis of phishing-email inspection beyond conventional AOI-based eye-tracking measures by introducing a complementary scene-based indicator of global visual exploration. AOI-based metrics capture where and for how long people look, though longer inspection does not necessarily reflect greater suspiciousness of the emails. Instead, scene-based metrics capture the overall structure of the exploration strategy (how systematically fixations are distributed across the stimulus space). Consistent with this interpretation, Wang et al. [12] argue that it is not the total amount of time spent on an email that improves phishing detection, but how effectively that time is used, including which deception indicators the user attends to. A non-suspicious email could elicit longer viewing time because participants are trying to interpret it within their own contextual frame, whereas a suspicious email could be labeled quickly as suspicious after only a minimal amount of time. In such cases, fixation duration could reflect interpretative effort rather than the intrinsic suspiciousness of the stimulus. Prior research similarly suggests that attention to phishing-related cues is not linearly associated with judgment of suspicion [4]. A complementary scene-based measure is therefore useful because it captures the overall spatial organization of fixations across the entire stimulus, rather than focusing only on areas of interest defined by the researcher.

A well-established candidate for this purpose is the Nearest Neighbor Index (NNI), a spatial statistics measure originally proposed by Clark and Evans to quantify spatial relationships in point patterns [13] and was later introduced into eye-movement research as an indicator of scanning strategy and has since been used across a range of Human Factors and HCI contexts to assess changes in global visual exploration associated with mental workload, including aviation, maritime scenarios, automotive driving, gaming tasks, and website exploration (see [14,15] for the most recent findings). Conceptually, the NNI compares the average observed distance between the nearest neighbors among fixations with the distance expected under complete spatial randomness. Values around 1 are consistent with spatial randomness; values less than 1 indicate clustered fixations, while values greater than 1 indicate a more regular and dispersed pattern. Calculated across all fixations within a defined reference area, the NNI provides a compact, AOI-independent description indicating whether visual sampling is concentrated in a few locations or distributed across the entire screen. This is particularly relevant for phishing detection, where potentially diagnostic clues may be distributed across both the header and body of the email, and where analyses based solely on the AOI might underestimate significant differences in how users navigate the stimulus while forming a judgment. The explicit theoretical baseline (NNI = 1) also makes the index intrinsically normalized and easily comparable across conditions and stimuli, allowing for an immediate directional interpretation of deviations. Consequently, especially in phishing detection, often studied using AOI-centric approaches where certain cues may be systematically overlooked, a global indicator such as the NNI can capture differences in scanning strategies that might not entirely emerge from local measures.

### 1.3. The Present Study

Accordingly, the present study focuses on the NNI as an index of visual exploration strategy during email classification, and explores the informational role of the NNI compared to other ocular and behavioral metrics (fixation duration and decision time). For the NNI, we test whether it differs between suspicious and non-suspicious emails, indicating that message type elicits different global exploration patterns, and between correct and incorrect trials, indicating that classification success is associated with a distinct spatial organization of fixations. In addition, we test whether the relationship between NNI and performance varies as a function of cybersecurity knowledge, consistent with the role of individual differences in phishing-related decision-making.

## 2. Materials and Methods

### 2.1. Participants

Thirty-two healthy volunteers (18 females, 14 males), aged between 18 and 40 years (*M* = 26.0; *SD* = 2.89), were recruited through word of mouth. Sample size was determined through MorePower 6.0.4 [16], with a statistical significance level of 0.05, a statistical power of 0.90, and an η^2^ of 0.06 for a 2 × 2 repeated-measures ANOVA with four levels. All the participants reported normal or corrected-to-normal vision, and were native Italian speakers. The study was approved by the CERT (Comitato Etico per la Ricerca Transdisciplinare, Sapienza University of Rome, protocol number CERT_18CEE1E772F) and conducted in accordance with the principles of the Declaration of Helsinki.

### 2.2. Instruments and Measures

Cybersecurity Awareness INventory (CAIN)*:* To assess users’ knowledge in the field of cybersecurity, the Italian version of the Cybersecurity Awareness Inventory [17] was used. The test consists of 30 TRUE/FALSE items, in which the participant tests their knowledge in the cybersecurity domain.

**Figure 1:**
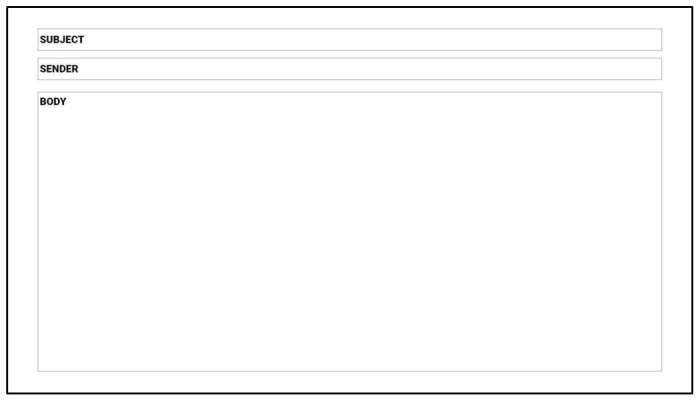
Wireframe of the stimuli layout.

Email selection: The final set of 106 emails was selected from the initial pool of 1016 messages using a stratified selection procedure designed to balance three main characteristics of the stimuli: topic category, message body length, and phishing cue profile. Emails were first classified by topic category (banking/finance, shipping/e-commerce, universities, healthcare, public institutions, leisure/discounts, miscellaneous, personal) and by message body length. Subsequently, suspected emails were selected to ensure a diverse representation of the predefined phishing cues, including sender-domain anomalies, suspicious subject-line features, unusual greetings, atypical requests, threats or promises of benefits, malicious links or attachments, grammatical or punctuation inconsistencies, and absent or generic signatures. The phishing emails were obtained from publicly available online repositories that archive real-world phishing campaigns and authentic phishing messages received directly by members of the research team. This sourcing strategy was adopted to ensure that participants were exposed to attack attempts that were circulating and broadly representative of common phishing tactics. Non-suspicious emails were sampled from messages received by the same research team and were selected to be comparable to phishing stimuli in terms of overall structure (Figure 1), message type, and format. The initial pool of 1016 emails was compiled and coded by one member of the research team and reviewed by a second member, who jointly defined the phishing cue criteria and applied them to classify each message. Selection disagreements were resolved through discussion and consensus. Formal inter-rater reliability indices were not computed for this process.

Suspicious and non-suspicious emails did not differ significantly either in character length, *t*(104) = 0.15, *p* = 0.884, or in word count, *t*(104) = 0.28, *p* = 0.784. Average mail length was balanced between suspicious emails (*M* = 524.90, *SD* = 382, 131–1990 characters) and non-suspicious emails (*M* = 514, *SD* = 266, 160–1253 characters).

Phishing emails were selected to systematically vary the presence of cues typically considered diagnostic in phishing email research [3,5,7]. The criteria used for stimulus coding and selection were the sender domain, subject line, and body text. Regarding the sender domain, emails could include domain manipulation like misspelling or typosquatting, such that the domain appeared visually similar to a legitimate or institutional domain, but contained minimal character-level alterations (e.g., substitutions, omissions, inversions). In other cases, the domain was clearly non-institutional or inconsistent with the sender identity. Regarding the subject line, the subject could include content designed to act as a salient stimulus (e.g., reward, urgency, authority, trust). In some stimuli, capitalization, emphatic punctuation, or special symbols were used to increase visual salience. Some emails also featured a missing subject (“-”). Regarding the body text, content could include generic or unusual greetings, atypical requests (e.g., money transfer, sending files, sharing sensitive information), threats of negative consequences for inaction or promises of immediate benefits, as well as potentially malicious links, requests to reply with confidential data, or suspicious attachments. Several messages included grammatical errors. Moreover, the signature could be absent or generic, lacking clear institutional references or contact information consistent with the claimed sender identity.

After coding phishing emails for the number of criteria present (from 1 to 8), stimuli were selected to ensure a balanced distribution across levels, with 4 phishing emails per level, yielding 32 selected emails. Given the total of 106 emails, the final set comprised 32 suspicious and 74 non-suspicious emails, presented as static stimuli during the classification task. The larger number of non-suspicious emails was intentionally adopted to approximate a more ecologically valid inbox context, in which legitimate messages are typically far more frequent than phishing attempts.

Behavioral Measures: To reduce the confounding effect of each participant’s personal interpretative context, participants were asked to judge whether emails were suspicious or not suspicious, rather than explicitly classify them as phishing attempts. This methodological choice was intended to orient participants toward evaluating the general suspiciousness of the message rather than their subjective plausibility of a phishing attempt. Responses were used to derive Signal Detection Theory outcome categories (Hit, Miss, False Alarm, Correct Rejection) by crossing the response with the true label of the stimulus [18]. Thus, the a priori reference criterion for correctness was the ground-truth classification of each stimulus (phishing vs. non-phishing), established before data collection. Namely, a phishing email classified as suspicious was labeled a “Hit”, while one classified as not suspicious was labeled a “Miss”. Conversely, a legitimate email classified as suspicious was labeled a “False Alarm”, while one classified as not suspicious was labeled a “Correct Rejection”. Decision time (seconds) was computed as the time elapsed from stimulus onset to the keypress indicating that a decision had been made.

Eye-tracking Measures: Eye movements were recorded binocularly, at a sampling rate of 150 Hz, using a Gazepoint GP3 HD infrared eye tracker (0.5–1° accuracy; 0.1 spatial resolution RMS; according to the manufacturer; Gazepoint Research Inc., Vancouver, BC, Canada) and proprietary software for stimulus presentation and data export. The device was positioned at the base of the display at approximately 60 cm from the participant. Raw gaze data were processed to obtain fixation-level coordinates (X, Y) and stimulus-level oculometric indicators following standard eye-tracking procedures [19,20]. To index spatial deployment of fixations, the visual exploration area was estimated as the convex hull area of fixation points, and workload-related spatial clustering was quantified via the Nearest Neighbor Index (NNI) [14], computed as the ratio between the observed mean distance between each fixation and its nearest neighboring fixation and the expected distance under complete spatial randomness.

NNI = d(NN)/d(ran)

where d(NN) is the observed mean nearest-neighbor distance between fixations and d(ran) is the expected mean nearest-neighbor distance under complete spatial randomness. Mean fixation duration during email exploration was also computed to provide a complementary measure of information processing. All oculometric indices were computed at the stimulus level and then averaged within participants as a function of experimental conditions (email type and response outcomes). For descriptive purposes, three Areas of Interest (AOIs) were defined on each email stimulus (Sender, Subject, and Body) to obtain the number of fixations per AOI.

### 2.3. Procedure

After agreeing to participate and signing the informed consent form and the information sheet regarding data processing, participants were assigned a unique code to ensure anonymity during the storage of behavioral data. Before the task, all participants completed the CAIN questionnaire in order to verify their level of knowledge of cyber threats. Before the beginning of the actual experimental task, a calibration procedure (2 min) was performed. The participants followed the 9 reference points shown on the screen with their gaze. In this phase, the experimenter checked: the correct framing of both eyes; adequate focus; and tracking quality (e.g., absence of prolonged signal loss). In cases where calibration proved insufficient, it was repeated. Calibration was considered acceptable when both eyes were correctly detected and the nine-point calibration produced stable gaze tracking across the screen, with no prolonged signal loss during the calibration check. During the task, efforts were made to minimize head movements and sources of disturbance (reflections, particularly reflective glasses, uncontrolled lighting), in order to reduce artifacts and missing samples. The task was performed in a silent room, illuminated by artificial light only. Participants were then presented, individually and in full screen (1920 × 1080; 23.8”), with 106 emails in static image format. For each trial, each participant had a maximum of 30 s to make a dichotomous decision about the nature of the message, indicating whether the observed email was authentic (non-suspicious) or a phishing attempt (suspicious). The instruction given to participants was: “For each email, decide whether the message appears suspicious or non-suspicious. Suspicious emails are those that, based on the information visible in the message, could plausibly represent a phishing attempt or an unsafe communication; non-suspicious emails are those that do not show such signs.” At the time of the decision, participants were instructed to press the SPACEBAR on the keyboard in front of them and communicate the outcome of the decision aloud to the experimenter, who was the only person besides the participant to be in the room for the entire duration of the task. After communicating the decision, participants could press the SPACEBAR again and proceed to the next email. The entire session lasted approximately 30 min. Participation in the study did not involve risks or discomfort for participants’ health. All participants were informed that they could interrupt the experiment at any time.

## 3. Data Analysis

*Preliminary data processing*: Response accuracy was computed by comparing the participant’s response to the true label of each email. Each trial was re-coded into SDT outcome categories (Hit, Miss, False Alarm, Correct Rejection) [18]. Trials with a very low number of fixations (below the 5th percentile of the fixation-count distribution) were excluded from subsequent analyses to reduce the influence of unreliable eye-tracking segments (e.g., signal loss or insufficient visual exploration) and to ensure that oculometric indices were computed from a minimum amount of gaze data. Fixation data were processed from the exported file in which each row corresponded to a fixation associated with a specific stimulus. The reference area required for the computation of the expected nearest-neighbor distance in the NNI was defined as the convex hull area of the cleaned fixation coordinates. The convex hull polygon was obtained via the monotone chain algorithm [21], and its area was computed using Gauss’s shoelace method. Trials with fewer than three distinct fixation points after cleaning were considered insufficient for a reliable convex-hull estimation. Accordingly, the convex hull area and NNI for that stimulus were considered as missing. Spatial clustering was summarized via the NNI as the ratio between the observed mean nearest-neighbor distance and the expected distance estimated from the reference area and the number of fixations [14]. Because the reference area used to compute the expected nearest-neighbor distance is derived from the same fixation set used to compute observed distances, NNI values should be interpreted as reflecting both the spatial arrangement of fixations and the extent of the explored area (as captured by the convex hull). This choice was adopted to let the reference area adapt to trial-by-trial differences in exploration, consistently with prior NNI applications in which the convex hull is used as an envelope for the fixation pattern. Fixation coordinates (X, Y) were used to assign each fixation to an AOI by comparing coordinates with predefined rectangular boundaries (Sender, Subject, Body), and to provide average fixation count for descriptive purposes. Data from one participant were excluded due to technical issues during recordings, and data from another participant were also excluded because eye fixations were not recorded for a large portion of the task. After excluding the two participants with substantial recording problems, 157 trials out of 3180 were removed based on the fixation-count threshold, corresponding to a trial-level data loss of approximately 4.9%. No additional participant- or trial-level quality thresholds were applied beyond the exclusion of participants with largely incomplete gaze recordings and the 5th-percentile fixation-count criterion.

*Descriptive analysis*: Descriptive statistics were computed for categorical variables (i.e., prior technical assistance due to viruses/security vulnerabilities, use of two-factor authentication) and for device-use variables. Each device category (desktop, laptop, tablet, smartphone, smartwatch) was coded as a binary indicator (0 = not used regularly; 1 = used regularly). A composite index of the number of devices used regularly was obtained by summing device indicators. Distributions and missing values were inspected prior to inferential analyses.

*Main analysis*: All analyses were conducted with Jamovi software (version 2.6). The normality assumptions were verified by observing asymmetry and kurtosis of the distributions of every single variable. Mean fixation duration variables showed departures from normality and were therefore log-transformed prior to inferential analyses. All ANOVA/ANCOVA models involving fixation duration were conducted on the log-transformed values, whereas descriptive statistics and figures are reported in the original scale to facilitate interpretation. (Tables S1 and S2). On data from the 30 analyzed participants, repeated-measures analyses of variance were conducted on the within-subject factors of interest. For the NNI, mean fixation duration, and decision time, 2 × 2 ANOVAs were performed with signal presence (suspicious versus non-suspicious emails) and outcome (right and wrong answers) as within-subject factors. These models were planned to test whether these metrics are capable of explaining participants’ exploration strategies across the four SDT outcomes (hits, correct rejections, misses, and false alarms). To study the role of knowledge in the cybersecurity domain, the same analyses were replicated in the form of repeated-measures ANCOVAs by introducing the CAIN score as a covariate. The continuous covariate was mean-centered prior to the repeated-measures ANCOVA to scale the intercept to the sample mean and to ensure an accurate and interpretable estimation of the within-subjects marginal means [22]. For all analyses, a significance level of alpha = 0.05 was adopted, and effect size indices (η^2^_p_) were reported. In case of statistical significance, comparisons between conditions were examined further through post hoc comparisons on estimated marginal means, with Bonferroni correction to control Type I error in multiple comparisons. Because all repeated-measures factors included only two levels, the sphericity assumption was automatically satisfied; therefore, Greenhouse-Geisser corrections were not required. To support the interpretation of NNI in relation to complete spatial randomness (NNI = 1), we additionally conducted exploratory post hoc two-tailed one-sample *t*-tests comparing the participant-level mean NNI in each condition against the theoretical value of 1 (H_a_: μ ≠ 1).

## 4. Results

*Sample characteristics*: In the analyzed sample (*N* = 30), participants reported using, on average, approximately 2.5 devices regularly (*M* = 2.50; *SD* = 0.73; 1–4). Overall, this suggests a predominantly multi-device usage profile. Specifically, device use was strongly dominated by smartphones and laptops. Overall, 96.7% of participants (*n* = 29) reported regular smartphone use, and 86.7% of them (*n* = 26) reported regular laptop use. By contrast, the use of desktop computers, tablets, and smartwatches was markedly less frequent. Regular desktop use was reported by 23.3% (*n* = 7), tablet use by 26.7% (*n* = 8), and smartwatch use by 16.7% (*n* = 5). With regard to prior technical assistance for problems related to viruses or security vulnerabilities, 66.7% of participants (*n* = 20) reported no prior request for assistance, whereas 33.3% (*n* = 10) reported that they had requested such assistance in the past. This indicates that roughly one-third of the sample had experienced security-related issues requiring external support. Concerning email access and two-factor authentication (2FA), the most frequent response was non-use of 2FA (53.3%, *n* = 16). A further 33.3% (*n* = 10) reported using 2FA, while 13.3% (*n* = 4) stated that they did not know what 2FA is. Taken together, these descriptive findings point to a relatively low uptake of 2FA and suggest the presence of a subgroup with limited awareness of this security measure. Concerning cybersecurity awareness, the average CAIN score was 26.47 (*SD* = 2.74).

*Task performance:* On average, the number of valid trials was 100.7 (SD = 7.75, 73–106). Descriptive statistics for the answers by outcome, across the 30 participants, showed the following distributions: Right Answers, *M* = 76.1 (*SD* = 10.55; 51–91), and Wrong Answers, *M* = 24.6 (*SD* = 7.52; 11–42). Descriptive statistics for the answers by outcome, using SDT levels [18], are reported in Table 1. Descriptive statistics for decision time showed the following distribution: *M* = 12.3 s (*SD* = 3.84; 5.77–20.5).

For trials excluded because fixation count was below the 5th percentile, the mean number of excluded trials per participant (N = 30) was: hits 1.80 (*SD* = 2.76; 0–13), misses 0.03 (SD = 0.18; 0–1), correct rejections 1.17 (*SD* = 1.98; 0–5), and false alarms 1.93 (*SD* = 4.45; 0–22).

**Figure 2:**
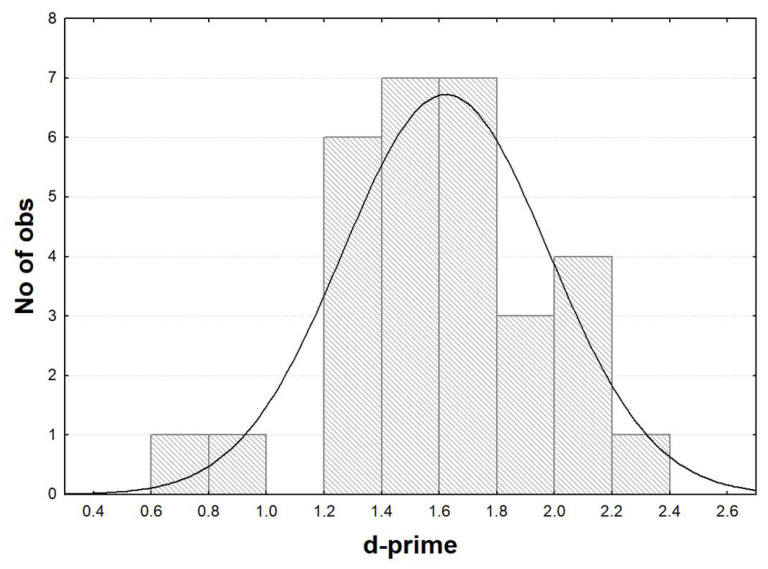
Distribution of *d*’ (*M* = 1.62, *SD* = 0.36).

**Figure 3:**
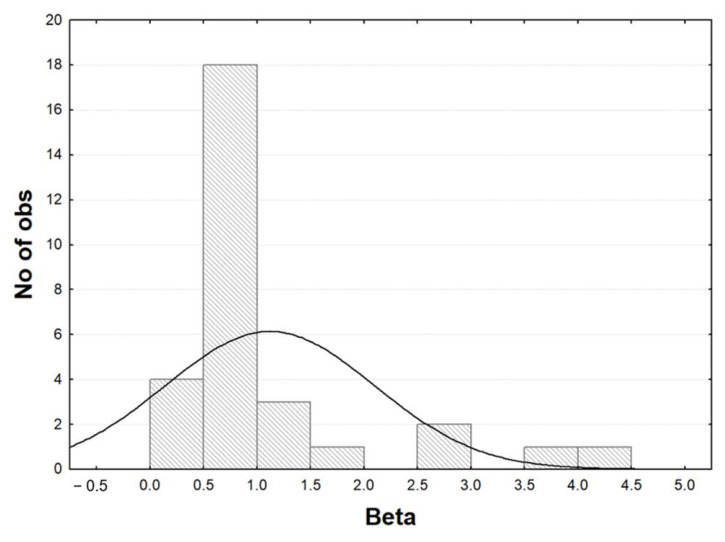
Distribution of *β* (*M* = 1.11, *SD* = 0.98).

D’ was calculated as the difference between the z-transformed hit rate and the z-transformed false alarm rate, and response bias (β) was calculated as the ratio between the height of the standard normal distribution at the z-transformed hit rate and at the z-transformed false alarm rate. The distributions of *d*’ and *β* in the sample are reported in Figure 2 and Figure 3, respectively.

To examine the relationship between CAIN scores and phishing-attempt detection, Pearson’s correlations were computed between mean-centered CAIN scores and signal-detection indices. Higher CAIN scores were significantly associated with a lower number of Misses, r(28) = −0.56, *p* = 0.001, and with a higher hit rate, r(28) = 0.47, *p* = 0.008. In contrast, correlations with d’, false-alarm rate, Hits, False Alarms, and Correct Rejections were not statistically significant (all *ps* > 0.05).

**Figure 4:**
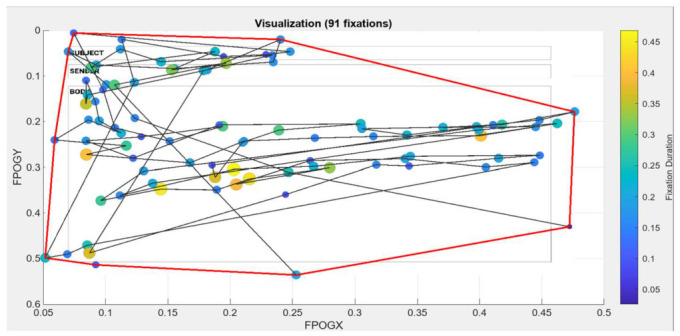
Example fixation distribution over an email stimulus. The black line represents the fixation path. The convex hull polygon (red line) defines the trial-specific reference area used to estimate the expected nearest-neighbor distance for NNI computation(see Section 3).

Descriptive statistics of gaze allocation and duration: Descriptive statistics for the number of fixations per AOI, across the 30 participants, valid trials were as follows: Number of fixations in Sender (*M* = 6.75; *SD* = 2.70; 2.83–13.5), Subject (*M* = 6.01; *SD* = 3.08; 1.35–14.1), and Body (*M* = 41.11; *SD* = 12.03; 16.38–64.6). Overall fixation distributions (Figure 4) were comparable across email types, with a mean of 51.6 fixations for suspicious emails (*SD* = 14.0; 30.9–84.5) and 54.9 fixations for non-suspicious emails (*SD* = 14.0; 34.0–85.4). Across response accuracy, participants produced fewer fixations when providing right answers (*M* = 52.6; *SD* = 13.0; 36.4–82.8), compared to wrong ones (*M* = 58.5; *SD* = 16.2; 30.4–92.6). Across Signal Detection Theory outcomes, mean fixation counts differed across response categories, with lower values for Hits (*M* = 49.3, *SD* = 12.9; 30.9–79.1) and progressively higher values for Correct Rejections (*M* = 55.0, *SD* = 14.2; 34.6–84.3) and False Alarms (*M* = 57.1, *SD* = 16.9; 30.6–94.0), peaking for Misses (*M* = 62.3, *SD* = 19.5; 29.9–103.7).

Main Results: After filtering, no missing values were detected for any of the variables. An examination of the descriptive statistics revealed, for the Decision Time variables, generally low values of skewness and a slight deviation from normality in the kurtosis of Decision Time in Misses (k = −1.057) alone; however, this value was retained as it was of modest magnitude. The variables related to mean fixation duration, on the other hand, exhibited higher indices of skewness and kurtosis; for this reason, they were subjected to logarithmic transformation in order to improve their adherence to normality. After logarithmic transformation, the mean fixation duration variables exhibited distributions that were generally more appropriate for the analyses, with low skewness and generally acceptable kurtosis; only mean fixation duration in correct rejection showed a slight increase in kurtosis (k = 1.193) (Table S2).

**Figure 5:**
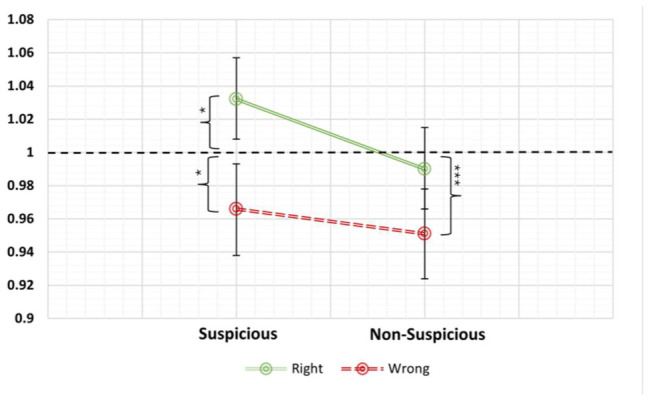
Estimated marginal means of NNI by Signal and Outcome (95% CI). The horizontal dashed line represents the baseline for spatial randomness (NNI = 1). Note: * *p* < 0.05; *** *p* < 0.001.

The repeated-measures ANOVA on NNI revealed a significant main effect of Outcome, *F*(1, 29) = 31.01, *p* < 0.001, η^2^_p_ = 0.517, and a significant main effect of Signal, *F*(1, 29) = 9.70, *p* = 0.004, η^2^_p_ = 0.250. Namely, NNI was higher for right answers and suspicious emails than for wrong answers and non-suspicious emails, respectively. Estimated marginal means showed that NNI was highest for Hits (*M* = 1.032, 95% CI [1.008–1.057]) and lowest for False Alarms (*M* = 0.951, 95% CI [0.924–0.978]) (Figure 5). The Outcome × Signal interaction was not significant, *F*(1, 29) = 2.10, *p* = 0.158, η^2^_p_ = 0.067. When the CAIN score was entered as a covariate in the model, the main effects of Outcome, *p* < 0.001, and Signal, *p* = 0.005, remained significant, and their interaction, *p* = 0.159 remained not significant. The effect of the CAIN score on NNI was not significant, *F*(1, 28) = 1.92, *p* = 0.177, η^2^_p_ = 0.064. Likewise, all the interactions involving the CAIN score were not significant (all *ps* > 0.05). In order to interpret NNI values relative to complete spatial randomness (NNI = 1), two-tailed one-sample *t*-tests were conducted within each email type and outcome. Given the four comparisons performed, statistical significance was evaluated using a Bonferroni-corrected alpha threshold of α = 0.0125 (0.05/4). In the one-sample *t*-test (H_a_: μ ≠ 1), it was observed that NNI in Hits significantly differed from 1, *t*(29) = 2.72, *p* = 0.011, Cohen’s *d* = 0.496, 95% CI [0.113–0.872], indicating a more dispersed/regular fixation pattern (NNI > 1), whereas NNI in Correct Rejection did not differ from 1, *t*(29) = −0.82, *p* = 0.421, *Cohen’s d =* −0.149, 95% CI [−0.508–0.212], suggesting a pattern consistent with spatial randomness. NNI in False Alarms was significantly lower than 1, *t*(29) = −3.76, *p* < 0.001, Cohen’s *d* = −0.687, 95% CI [−1.081–−0.283], indicating clustering of fixations. By contrast, although NNI in Misses was lower than 1, this comparison did not survive Bonferroni correction, *t*(29) = −2.53, *p* = 0.017, Cohen’s *d* = −0.461, 95% CI [−0.835–−0.081].

**Figure 6:**
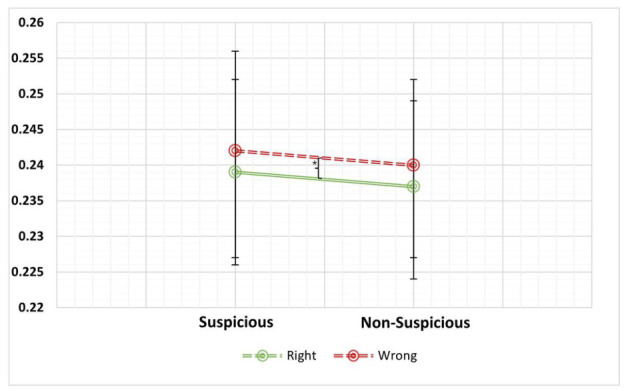
Estimated marginal means of mean fixation duration (in seconds) by Signal and Outcome (95% CI). Note. * *p* < 0.05.

The repeated-measures ANOVA on mean fixation duration showed a significant main effect of Outcome, *F*(1, 29) = 6.97, *p* < 0.013, η^2^_p_ = 0.194. Specifically, right answers showed a lower mean fixation duration than wrong answers. Estimated marginal means showed lower fixation durations for Hits (*M* = −0.625, 95% CI [−0.648–−0.603]) and Correct Rejections (*M* = −0.630, 95% CI [−0.651–−0.609]) than for Misses (M = −0.622, 95% CI [−0.646–−0.597]) and False Alarms (*M* = −0.624, 95% CI [−0.645–−0.603]) (Figure 6). The main effect of Signal showed a trend toward significance, *F*(1, 29) = 3.03, *p* = 0.093, η^2^_p_ = 0.094; mean fixation duration was higher when the signal was present. However, the Signal × Outcome interaction was not statistically significant, *F*(1, 29) = 0.260, *p* = 0.614, η^2^_p_ = 0.009. When the CAIN score was entered as a covariate, the main effect of Outcome remained significant, *p* = 0.011, and the main effect of Signal remained not significant, *p* = 0.093, as did their interaction, *p* = 0.616. The effect of the CAIN score on mean fixation duration was not significant, *F*(1, 28) = 0.13, *p* = 0.72, η^2^_p_ = 0.005. Likewise, all the interactions involving the CAIN score were not significant (all *ps* > 0.05).

**Figure 7:**
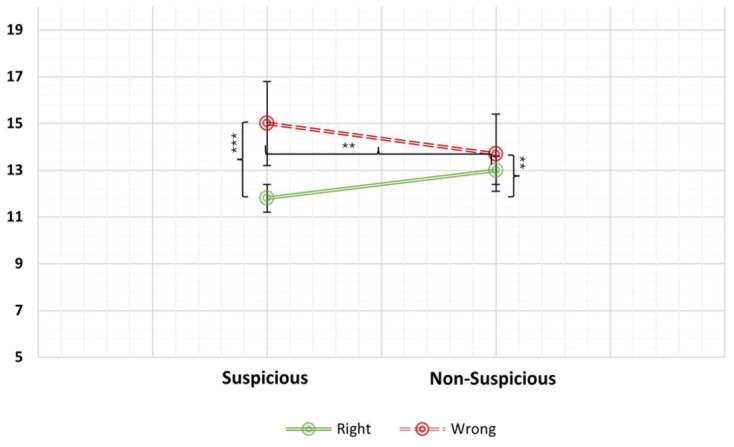
Estimated marginal means of decision time (in seconds) by Signal and Outcome (95% CI). Note. ** *p* < 0.01; *** *p* < 0.001.

The repeated-measures ANOVA on decision time showed a significant main effect of Outcome, *F*(1, 29) = 44.43, *p* < 0.001, η^2^_p_ = 0.605. The main effect of Signal was not significant, *F*(1, 29) = 0.01, *p* = 0.924, η^2^_p_ = 0.000. However, the Signal × Outcome interaction was significant, *F*(1, 29) = 12.49, *p* < 0.001, η^2^_p_ = 0.301. Bonferroni-corrected post hoc comparisons on the estimated marginal means indicated that decision time was significantly shorter for correct responses when the phishing signal was present (Hit; *M* = 11.8 s, 95% CI [10.5–13.2]) compared with incorrect responses, both when the signal was present (Miss; *M* = 15.0 s, 95% CI [13.2–16.8]), *p* < 0.001, and when it was absent (False Alarm; *M* = 13.7 s, 95% CI [12.1–15.4]), *p* = 0.001 (Figure 7). A trend toward faster responses was also observed for Hits relative to correct responses when the signal was absent (Correct Rejection; *M* = 13.0 s, 95% CI [11.6–14.4]), though this difference did not reach significance, *p* = 0.071. In addition, decision time was significantly shorter for correct rejections than misses, *p* = 0.006. When the CAIN score was entered as a covariate in the model, the main effect of Outcome remained significant, *p* < 0.001, whereas the main effect of Signal, *p* = 0.922, was not significant and the Outcome × Signal interaction, *p* = 0.002, remained significant. The effect of the CAIN score on decision time was not significant, *F*(1, 28) = 2.67, *p* = 0.113, η^2^_p_ = 0.087. Likewise, all the interactions involving the CAIN score were not significant (all *ps* > 0.05).

## 5. Discussion

This study extends eye-tracking research on phishing detection by introducing a scene-based measure of global visual exploration, the Nearest Neighbor Index (NNI). Unlike classical measures, NNI captures whether fixations are clustered or more evenly distributed across the stimulus, without depending on pre-defined regions of interest.

At the aggregate level, the main findings show that NNI was higher for suspicious than for non-suspicious emails, suggesting a shift in exploration strategy when the stimulus imposes greater task demands, and higher for correct than for incorrect classifications, suggesting that errors could be associated with visual anchoring on a limited subset of elements, possibly salient but not diagnostic. Indeed, NNI values in false alarms were significantly lower than 1, whereas misses showed a similar clustering tendency that did not survive Bonferroni correction. Crucially, hits but not correct rejections showed NNI values significantly higher than 1, indicating a more dispersed fixation pattern for correct responses only when the phishing signal is present. Accordingly, NNI may provide an indirect index of differences in visual exploration strategy. When a phishing signal is present, dispersed fixations may reflect a broader and more systematic cue-checking strategy, increasing the probability that diagnostic elements are sampled and correctly interpreted. Conversely, the more clustered pattern observed descriptively in misses may reflect a narrower exploration focus, reducing the likelihood that phishing-relevant cues are included within the inspected region. When no phishing signal is present, fixation patterns consistent with spatial randomness appear to be associated with correct rejections, whereas clustered fixations are associated with false alarms. In this case, a narrow focus may increase the likelihood of overinterpreting salient but non-diagnostic elements as suspicious. Thus, classification errors appear more likely when the stimulus is explored less broadly, although only correct detection in the presence of a phishing signal is associated with a clearly broader visual exploration strategy.

A possible interpretation of these findings lies in the fact that, in the literature, the NNI has been linked to task demands. Indeed, the NNI is interpreted in relation to workload: as workload increases, the NNI tends to indicate more distributed gaze patterns (less concentrated on a few points) [14]. This higher dispersion is interpreted as an adaptation strategy: rather than keeping attention tightly focused, the observer broadens visual sampling to maximize readiness and quickly intercept potentially relevant incoming information. Concurrent bottom-up processes allow for salient elements in the email to quickly capture attention and then trigger a broader exploration of the entire scene, which overall increases the dispersion captured by the NNI [14]. This interpretation could explain the high NNI for right answers when a phishing cue is present (Hits), even though the lowest decision time was observed in this condition.

Accordingly, even though only marginally significant, suspicious emails were associated with higher mean fixation durations [23,24].

Importantly, the interpretability of these gaze dynamics is strengthened by the fact that NNI is anchored to an explicit theoretical benchmark (NNI = 1), corresponding to spatial randomness. This reference point allows changes in exploration to be described not only as relative differences between conditions, but as meaningful departures from (or adherence to) a common baseline: values below 1 reflect increasingly clustered, spatially anchored sampling, whereas values above 1 reflect an expansion toward more distributed coverage of the email. In the present data, this anchor makes it possible to frame error patterns as a contraction of exploration, particularly for false alarms, with misses showing a descriptively similar tendency, while correct performance, particularly when a phishing cue is present, reflects a broadening of sampling (hits above 1), and correct rejections remain closer to a randomness-consistent pattern.

Although decision time did not differ significantly between suspicious and non-suspicious emails overall, participants responded faster when they were correct than when they were incorrect, and hits were faster than both misses and false alarms. This pattern is consistent with the idea that longer response times in incorrect trials could reflect uncertainty and prolonged search that remains poorly organized, as suggested by the clustered scanning observed in misses and false alarms, rather than more effective processing that resolves ambiguity successfully. Accordingly, mean fixation duration was lower for correct than incorrect answers, reflecting prolonged or inefficient stimulus processing [23,24].

Crucially, conventional intensity-based indices (e.g., decision time, number of fixations) do not necessarily map onto the informativeness of visual inspection. Fewer fixations or faster decisions can reflect superficial processing, but they can also reflect a more efficient strategy in which a limited number of fixations is strategically deployed to sample a wider portion of the email and intercept diagnostic cues. Because the NNI indexes the spatial distribution of fixations rather than their quantity, it can reveal “broad-but-efficient” exploration that would otherwise be misclassified as reduced or incomplete inspection. In this sense, NNI provides a complementary, and in some situations more sensitive, indicator of information acquisition, showing whether the available fixations were concentrated on a narrow subset of elements or distributed to maximize coverage of potentially relevant regions.

The distribution of signal detection indices revealed a dissociation between sensitivity and decision criterion. Specifically, d′ values were relatively compact and approximately unimodal, clustering around moderate to high levels of discrimination, suggesting that participants shared a broadly similar ability to distinguish between signal and noise. In contrast, β values showed a markedly skewed and potentially multimodal distribution, with a concentration around neutral to moderately liberal criteria and a long tail toward highly conservative responding. This pattern could indicate substantial variability in decision strategies despite comparable perceptual sensitivity. Moreover, this variability in β could reflect systematic individual differences in how participants regulate their responses under uncertainty. Future research, ideally with larger samples, should further investigate this aspect by identifying and characterizing subgroups of participants exhibiting distinct decision strategies.

Associations between CAIN scores and SDT outcomes suggest that higher CAIN scores were associated with a reduced tendency to miss phishing attempts and with a higher hit rate, although they were not significantly related to overall discrimination performance indexed by d’.

At the same time, CAIN did not emerge as a consistent predictor across models, suggesting that cybersecurity knowledge may not directly shape the moment-by-moment spatial organization of gaze during visual search. Instead, CAIN may be more closely associated with later-stage processes, such as the interpretation of suspicious cues once they have been encountered, rather than with how visual exploration is spatially organized during inspection—a dimension that NNI appears to better capture.

Taken together, these findings are consistent with the view that cybersecurity knowledge represents a potentially relevant individual-difference factor, though they do not support strong causal claims regarding its direct influence on online gaze behavior. From an applied standpoint, these results tentatively suggest that training interventions might benefit from integrating knowledge-based instruction with approaches aimed at supporting more systematic visual inspection of email content. These observations should, however, be interpreted with caution: the modest sample size (N = 30) limits statistical power to detect subtle covariate effects and higher-order interactions, and the generalizability of the findings to more ecologically valid phishing-detection contexts remains to be established. Further research with larger and more diverse samples is needed to clarify the role of individual differences in shaping gaze-based indicators of phishing detection.

## 6. Limitations and Future Directions

The article also highlights methodological issues that may limit interpretation in controlled phishing studies, especially priming effects and reduced ecological validity when participants know in advance that phishing messages are present. Prior work cited in the article has recommended more naturalistic designs, such as embedding phishing emails within broader email-management tasks. In addition, the relatively small sample size may have limited the statistical power of the covariate analyses involving CAIN, particularly for detecting smaller individual-difference effects. Relatedly, the sample consisted mainly of young adults within a restricted age range (18–40 years), which may limit the generalizability of the findings to older, less digitally experienced, or more heterogeneous populations. In the present study, stimuli were presented as static images within an explicit classification task, a format that may influence both decision criteria and scan behavior. Because participants were explicitly engaged in a suspiciousness-classification task, demand characteristics and heightened vigilance may also have influenced both their decisions and gaze patterns. A further methodological limitation concerns the coding of phishing cues used to guide stimulus selection and balancing. Phishing emails were coded for the presence of eight predefined cue categories (sender-domain anomalies, subject-line features, unusual greetings, atypical requests, threat or benefit appeals, malicious links or attachments, grammatical inconsistencies, and absent or generic signatures), but formal inter-rater reliability indices were not calculated for this procedure. Because cue classification served mainly as a stimulus-balancing criterion rather than as an outcome measure, its direct impact on the primary analyses is likely limited. Nonetheless, the lack of documented coder agreement reduces the transparency and replicability of the stimulus construction process.

Future studies should include explicit inter-rater reliability assessments when systematically coding phishing cue profiles. Future research should therefore examine whether NNI-based signatures of successful and unsuccessful detection generalize to more ecologically valid settings, such as real email clients, mixed-task environments, and more realistic base rates of phishing. It will also be important to test whether strategy-focused training or attention-guiding interface cues can causally reduce the clustered exploration patterns associated with misses and false alarms. Future work may also compare NNI with alternative global descriptors of gaze organization (e.g., spatial/visual entropy) to test convergent and discriminant validity across tasks and interfaces; for instance, Maggi and Di Nocera [17] reported that entropy rate and NNI show a similar pattern over time.

## 7. Conclusions

In conclusion, this study suggests that the detection of phishing emails depends not only on the amount of visual inspection, but on how gaze is distributed across the message. The Nearest Neighbor Index showed that successful detection is associated with broader and more regular visual exploration, whereas misses and false alarms are associated with clustered scanning. These findings indicate that global scene-based measures could complement traditional AOI-based metrics by revealing whether users inspect an email in a way that maximizes the probability of detecting diagnostic cues. More broadly, the results highlight the importance of visual exploration strategy in phishing detection and support the development of training and interface solutions aimed at fostering wider and more informative sampling of email content.

## Figures and Tables

**Table 1:** SDT outcomes (mean and standard deviation).

HITS	FALSE ALARMS
24.33 (3.99)	18.87 (9.30)
MISSES	CORRECT REJECTIONS
5.70 (2.96)	51.77 (11.04)

## Data Availability

The raw data supporting the conclusions of this article will be made available by the authors on request.

## Supplementary Materials

The following supporting information can be downloaded at https://www.mdpi.com/article/10.3390/jemr19030060/s1, Table S1: Descriptive statistics; Table S2: Logarithmic correction.

## References

[1] Anti-Phishing Working Group Phishing Activity Trends Report: 4th Quarter 2025. https://docs.apwg.org/reports/apwg_trends_report_q4_2025.pdf.

[2] Baltuttis D., Teubner T. (2024). Effects of Visual Risk Indicators on Phishing Detection Behavior: An Eye-Tracking Experiment. Comput. Secur..

[3] Ribeiro L., Sousa Guedes I., Cardoso C.S. (2024). Eyes on Phishing Emails: An Eye-Tracking Study. J. Exp. Criminol..

[4] McAlaney J., Hills P.J. (2020). Understanding Phishing Email Processing and Perceived Trustworthiness through Eye Tracking. Front. Psychol..

[5] Pietrantonio F., Botta A., Ventre G., Gallo L., Zinno S., Mancuso L., Presta R. Investigating Gaze Behavior in Phishing Email Identification. Proceedings of the 2023 7th Network Traffic Measurement and Analysis Conference (TMA).

[6] Thomopoulos G.A., Lyras D.P., Fidas C.A. (2024). A Systematic Review and Research Challenges on Phishing Cyberattacks from an Electroencephalography and Gaze-Based Perspective. Pers. Ubiquitous Comput..

[7] Pfeffel K., Ulsamer P., Müller N.H. (2019). Where the User Does Look When Reading Phishing Mails-An Eye-Tracking Study. Lecture Notes in Computer Science.

[8] Oh S., Bae Y., Thompson I.A., Im Y. (2025). Uncovering the Dark Patterns of Phishing Emails: An Eye-Tracking Analysis. IEEE Access.

[9] Zhuo S., Biddle R., Recomendable J.D., Russello G., Lottridge D. (2024). Eyes on the Phish(er): Towards Understanding Users’ Email Processing Pattern and Mental Models in Phishing Detection. Proceedings of the 2024 European Symposium on Usable Security (EuroUSEC 2024).

[10] Bergstrom J.R., Schall A.J. (2014). Eye Tracking in User Experience Design.

[11] Huang L., Jia S., Balcetis E., Zhu Q. (2022). ADVERT: An Adaptive and Data-Driven Attention Enhancement Mechanism for Phishing Prevention. IEEE Trans. Inf. Forensics Secur..

[12] Wang J., Li Y., Rao H.R. (2017). Coping Responses in Phishing Detection: An Investigation of Antecedents and Consequences. Inf. Syst. Res..

[13] Clark P.J., Evans F.C. (1954). Distance to Nearest Neighbor as a Measure of Spatial Relationships in Populations. Ecology.

[14] Maggi P., Di Nocera F. (2021). Sensitivity of the Spatial Distribution of Fixations to Variations in the Type of Task Demand and Its Relation to Visual Entropy. Front. Hum. Neurosci..

[15] Serra G., De Falco F., Maggi P., De Piano R., Di Nocera F. (2022). Website Complexity and Usability: Is There a Role for Mental Workload?. Int. J. Hum. Factors Ergon..

[16] Campbell J.I., Thompson V.A. (2012). MorePower 6.0 for ANOVA with Relational Confidence Intervals and Bayesian Analysis. Behav. Res. Methods.

[17] Di Nocera F., Tempestini G., Presaghi F. (2025). Reliability and Validity of the Cybersecurity Awareness INventory (CAIN). Behav. Inf. Technol..

[18] Macmillan N.A., Creelman C.D. (1991). Detection Theory: A User’s Guide.

[19] Holmqvist K., Nyström M., Andersson R., Dewhurst R., Jarodzka H., Van de Weijer J. (2011). Eye Tracking: A Comprehensive Guide to Methods and Measures.

[20] Duchowski A.T. (2017). Eye Tracking Methodology: Theory and Practice.

[21] Andrew A.M. (1979). Another Efficient Algorithm for Convex Hulls in Two Dimensions. Inf. Process. Lett..

[22] Aiken L.S., West S.G., Reno R.R. (1991). Multiple Regression: Testing and Interpreting Interactions.

[23] Liu J.C., Li K.A., Yeh S.L., Chien S.Y. (2022). Assessing Perceptual Load and Cognitive Load by Fixation-Related Information of Eye Movements. Sensors.

[24] Callan D.J. (1998). Eye Movement Relationships to Excessive Performance Error in Aviation. Proceedings of the Human Factors and Ergonomics Society Annual Meeting.

