# Supplementary material for: The Geometry of Suspicion: Visual Exploration Patterns in Email Phishing Detection
Source: J Eye Mov Res. 2026 Jun 1;19(3):60. doi: 10.3390/jemr19030060 (PMC13302592; doi:10.3390/jemr19030060)
Supplement: Supplementary file 1 [file jemr-19-00060-s001.zip › jemr-4336590-supplementary.pdf]

**Table S1.** Descriptive statistics.

| Variable* | N | Missing | Mean | Median | SD | Min | Max | Skewness | SE | Kurtosis | SE |
|---|---|---|---|---|---|---|---|---|---|---|---|
| H_DecTime | 30 | 0 | 11.811 | 10.860 | 3.608 | 6.605 | 19.456 | 0.863 | 0.427 | -0.061 | 0.833 |
| CR_DecTime | 30 | 0 | 13.010 | 12.913 | 3.742 | 7.400 | 21.283 | 0.434 | 0.427 | -0.390 | 0.833 |
| FA_DecTime | 30 | 0 | 13.715 | 13.547 | 4.403 | 6.354 | 22.621 | 0.412 | 0.427 | -0.317 | 0.833 |
| M_DecTime | 30 | 0 | 14.992 | 15.114 | 4.852 | 6.022 | 23.663 | 0.059 | 0.427 | -1.057 | 0.833 |
| H_NNI | 30 | 0 | 1.032 | 1.033 | 0.065 | 0.879 | 1.134 | -0.665 | 0.427 | 0.532 | 0.833 |
| CR_NNI | 30 | 0 | 0.990 | 0.988 | 0.066 | 0.844 | 1.124 | -0.210 | 0.427 | -0.076 | 0.833 |
| FA_NNI | 30 | 0 | 0.951 | 0.954 | 0.072 | 0.811 | 1.102 | 0.060 | 0.427 | -0.333 | 0.833 |
| M_NNI | 30 | 0 | 0.966 | 0.961 | 0.074 | 0.838 | 1.121 | 0.248 | 0.427 | -0.379 | 0.833 |
| H_FixD | 30 | 0 | 0.239 | 0.232 | 0.035 | 0.187 | 0.346 | 1.187 | 0.427 | 1.888 | 0.833 |
| CR_FIxD | 30 | 0 | 0.237 | 0.233 | 0.033 | 0.190 | 0.342 | 1.336 | 0.427 | 2.484 | 0.833 |
| FA_FIxD | 30 | 0 | 0.240 | 0.236 | 0.033 | 0.192 | 0.332 | 1.135 | 0.427 | 1.181 | 0.833 |
| M_FixD | 30 | 0 | 0.242 | 0.236 | 0.039 | 0.194 | 0.352 | 1.251 | 0.427 | 1.413 | 0.833 |

* Decision time in Hits (H_DecTime), Correct Rejections (CR_DecTime); False Alarms (FA_DecTime) and Misses (M_DecTime); Nearest Neighbour Index in Hits (H_NNI), Correct Rejections (CR_NNI), False Alarms (FA_NNI) and Misses (M_NNI); Mean fixation duration in Hits (H_FixD), Correct Rejections (CR_FixD), False Alarms (FA_FixD) and Misses (M_FixD).

**Table S2.** Logarithmic correction.

| Variable | N | Missing | Mean | Median | SD | Min | Max | Skewness | SE | Kurtosis | SE |
|---|---|---|---|---|---|---|---|---|---|---|---|
| H_FixD_LOG | 30 | 0 | -0.625 | -0.634 | 0.060 | -0.727 | -0.461 | 0.756 | 0.427 | 0.774 | 0.833 |
| CR_FixD_LOG | 30 | 0 | -0.630 | -0.633 | 0.057 | -0.721 | -0.466 | 0.898 | 0.427 | 1.193 | 0.833 |
| FA_FixD_LOG | 30 | 0 | -0.624 | -0.628 | 0.056 | -0.717 | -0.479 | 0.809 | 0.427 | 0.460 | 0.833 |
| M_FixD_LOG | 30 | 0 | -0.622 | -0.627 | 0.065 | -0.713 | -0.453 | 0.901 | 0.427 | 0.460 | 0.833 |
